# Increased arterial stiffness is associated with poorer social cognition in older age

**DOI:** 10.1038/s41598-025-86423-y

**Published:** 2025-01-17

**Authors:** Sarah A. Grainger, Tom G. Bailey, Natalie K. Vear, Jessica K. Mead, Xanthia E. Bourdaniotis, Jeff S. Coombes, Jenna L. Taylor

**Affiliations:** 1 School of Psychology, The University of Queensland, St Lucia, QLD 4072 Australia; 2 School of Human Movement and Nutrition Sciences, The University of Queensland, St Lucia QLD, Australia; 3 School of Nursing, Midwifery and Social Work, The University of Queensland, St Lucia QLD, Australia

**Keywords:** Theory of mind, Vascular aging, Arterial stiffness, Pulse wave velocity, Psychology, Ageing

## Abstract

It is now well established that vascular aging is a significant predictor of cognitive decline in older age. But what remains less clear is the role that vascular health plays in social cognitive aging. Therefore, we aimed to provide the first test of the relationship between arterial stiffness and theory of mind (ToM) in late adulthood. In a sample of 50 healthy older adults (Age: *M =* 70.08, *SD =* 3.93), we measured arterial stiffness via carotid-femoral pulse wave velocity and social cognition using two well validated measures of ToM (RMET, TASIT). The results revealed that arterial stiffness was a significant predictor of ToM performance when indexed via the RMET and the TASIT, accounting for 11% and 9% of unique variance in scores, respectively. These findings add to the broader literature showing that arterial stiffness is a key predictor of cognitive aging and show that this relationship extends to the domain of social cognition.

Social cognitive function refers to our capacity to detect, understand and respond to social cues. Social cognitive abilities are important for developing and maintaining social networks^1^, and therefore have direct implications for social connectedness^2^. Indeed, maintaining strong social networks is important for health and wellbeing at all stages of the lifespan^3^, but this is particularly the case in older age. Social isolation and loneliness have been consistently linked to a higher mortality risk^4–6^, and several studies have now shown that people who are less socially connected in older age are more likely to experience cognitive decline and even dementia^7,8^. Social cognitive impairments are also a prominent feature in many clinical disorders^9^. It is therefore concerning that there is a large literature showing that some aspects of social cognition change with normal aging^10–12^. Theory of Mind (ToM) refers to our capacity to understand the mental and emotional states of others, and to appreciate that these may differ from our own. ToM can be broken down into affective and cognitive components^13^. Whereas affective ToM refers to the ability to detect and understand others’ emotions and feelings, cognitive ToM refers to our capacity to understand belief states, desires and intentions. Several studies have now shown that both cognitive and affective ToM decline with normal aging^10,12,14,15^. Given the negative implications for broader social wellbeing, an important focus now is understanding the mechanisms that might explain these age-related changes so that targeted interventions and preventative strategies can be developed and implemented.

 It is well established that poor vascular health is linked to cognitive decline and a greater risk of dementia^16–19^. However, less research has focused on the role of vascular health in older adults’ social cognitive abilities. This is surprising given that vascular markers, such as arterial stiffness, have been linked to executive function impairments^20^, and critically, that some of the processes underlying executive function and ToM overlap^10,21^. Indeed, prior work has shown that age-related changes in ToM are associated with declines in executive control^15,22^. Neuroimaging studies have also shown that ToM and executive function rely on some shared neural substrates^23,24^. While vascular health can also be expressed as the presence of vascular disease or vascular function, the key focus of this study is the potential role that age-related structural vascular alterations might play in social cognitive change in late adulthood.

Arterial stiffening is characterized by a loss of elasticity in the artery walls, which reduces the artery’s capacity to expand and contract to deliver adequate blood flow to the brain^25^. Moreover, stiffness impairs the buffering capacity of the arteries, exposing the brain to increased pulsatile stress leading to cerebral small vessel disease and white matter damage^26,27^. Not surprisingly arterial stiffness has been coined the hallmark of vascular aging^28^ and has been consistently linked to cognitive decline and dementia in older adults^16,20^. Importantly though, the rate of arterial stiffness can be slowed by adopting healthy lifestyle choices^29,30^, which means that any adverse effects on brain health due to artery stiffness may be somewhat preventable. 

Surprisingly, no studies to date have directly measured arterial stiffness in relation to social cognition in older adults. Fischer et al. measured pulse pressure, which is an estimate of vascular risk derived from blood pressure and is often used as a surrogate for arterial stiffness^31,32^. Across two separate studies, they found that higher pulse pressure was associated with poorer cognitive ToM in healthy older adults. They additionally measured affective ToM in their second study but failed to find any relationship with pulse pressure. However, while pulse pressure is a simple proxy for arterial stiffness, it can be influenced by other factors, such as large-artery compliance, stroke volume, and reflected pressure waves^33,34^. The non-invasive gold standard for assessing arterial stiffness recommended by the American Heart Association is carotid-femoral pulse wave velocity (PWV)^34^, which measures the precise rate at which the arterial pulse travels through the artery, with a faster PWV being indicative of greater stiffness^35^.

The overarching aim of this study was to provide the first assessment of the relationship between PWV and social cognition in a group of healthy older adults. In line with the broader literature on arterial stiffness and cognition, and Fischer et al. who found that pulse pressure was associated with theory of mind^31,32^, we hypothesized that there would be a significant inverse relationship between PWV and ToM.

## Method

### Participants

We conducted a power analysis to determine the minimum number of participants required to detect a moderate effect size (*f*= 0.17, see^36^ with 80% power using hierarchical linear regression with age, sex, education, and executive function entered at Step 1 and PWV at Step 2. This revealed that we needed a minimum of 49 participants.

Participants were recruited from the local Brisbane community, and were excluded if they had a history of cardiovascular or cerebrovascular disease, a diagnosis of diabetes or uncontrolled hypertension, a neurological or psychiatric condition, or an orthopedic condition restricting the ability to exercise. All participants were screened for dementia using the Mini-Addenbrookes Cognitive Examination (Mini-ACE^37^, and were required to score above the cut-off of 21/30 in order to be included in the study. Participants were also screened for high blood pressure on the day of testing and were excluded if they recorded a reading > 140/>90mmHg. We initially recruited 59 participants for this study but seven were excluded based on the above criteria and two were excluded due to COVID restrictions in place at the time of testing. This project was approved by the Human Research Ethics Committee (HREC) at The University of Queensland. Data collection was completed between January 2020 and July 2021 in Brisbane, Australia. All participants provided written informed consent prior to taking part in the study and were compensated $80 in gift cards for their participation. This study was performed in accordance with the Declaration of Helsinki.

### Measures

**Reading the Mind in the Eyes Test (RMET).**The RMET is one of the most widely used measures of affective ToM available^38^. It includes 36 images depicting the eye region of faces showing different mental states. Participants are required to view each image and select the mental state that best describes what the person is feeling from a list of four options. Responses were either scored correct or incorrect, and a percentage accuracy score was calculated with the total number of correct trials. The RMET has been used extensively in older adult populations and is sensitive in detecting age-related changes in theory of mind^12^.

**The Awareness of Social Inference Test (TASIT).**The TASIT is another well validated measure of cognitive ToM^39^. We used Part 3 of the TASIT which measures an individual’s ability to detect white lies and sarcasm from brief videos. This task included 16 trials in total. After viewing each video, participants are asked four questions that enquire about the main protagonist (i.e., what they are doing, saying, thinking and feeling). All responses are made by selecting ‘yes’, ‘no’ or ‘don’t know’ and each question is scored correct or incorrect. Answers were converted into a percentage accuracy score. The TASIT has been used extensively in healthy older adult populations (see^10,14^).

**Trial Making Test.**The Trail Making Test is a neuropsychological assessment used to index cognitive flexibility, attention, and executive functioning. Part A of the test requires participants to connect a series of numbered circles starting from 1 and ending at 25. Part B requires participants to connect a series of circles but they must alternate between numbers and letters (e.g., 1 -A −2- B-3- C). The time to complete the task is recorded in seconds, with a faster score suggesting of better cognitive functioning. To index executive function, we calculated the difference score by subtracting Part A performance from Part B^40^.

**Vascular Assessments.**Participants attended the laboratory following an overnight fast, and had refrained from caffeine, alcohol, nicotine, and exercise on the morning of the testing session. They were assessed in a dark, temperature controlled room after resting in a supine position for 10 min. Blood pressure, heart rate, and carotid-femoral PWV were measured in accordance with recommended guidelines^34^ using SphygmoCor XCEL (AtCor Medical, West Ryde, NSW, Australia). For PWV, carotid pulse waves were measured by a hand-held tonometer with femoral pulse waves collected simultaneously with a thigh cuff. Blood pressure, pulse pressure, and heart rate were averaged from 3 measurements. PWV was analyzed according to recommended guidelines using the mean of two measurements within 0.5 m/s, or the median value if a third value was required.

## Procedure

This study was part of a larger testing protocol that included a graded exercise test, muscular strength tests, blood sampling, and other neurocognitive assessments^41^. Participants completed two separate testing sessions within 4 weeks of each other. In the first session, participants provided written informed consent and then completed screening assessments (Mini-ACE, blood pressure). Next, they completed a demographic questionnaire followed by a battery of cognitive and social cognitive tasks in a counterbalanced order. Participants were given verbal and written instructions for each task and were given as much time as they needed to complete the tasks. In the second session, participants’ blood pressure was measured first, followed by arterial stiffness via pulse wave velocity. All participants were debriefed and compensated at the end of the second session.

### Data Analysis

The relationship between arterial stiffness and social cognition was firstly examined through bivariate correlations between PWV and social cognition variables (RMET and TASIT). This was followed by hierarchical linear regression analyses to examine the degree to which arterial stiffness explained unique variance in social cognition performance (separately for TASIT and RMET). Given that executive function is related to ToM performance and arterial stiffness^20,42^, we included it in the analysis at Step 1 along with age, sex, education and BMI. This was followed by PWV at Step 2. Statistical analyses were performed in SPSS Statistics (IBM, version 29). Alpha was set at *p* < .05.

## Results

Sample characteristics including participant demographics, executive function, social cognition task performance as well as PWV are reported in Table 1.

**Table 1:** Sample characteristics.

Measure	*n*	Range	Mean (SD)	Total %
Age (years)	50	60–76	70.1 (9.5)	
Sex	50			56% Female
Education (years)	50	7–20	14.8 (3.1)	
BMI	50	19.1–37.8	25.9 (3.8)	
Current medication use	50			22%
Brachial systolic BP	50	105–149	127.82 (11.52)	
Brachial diastolic BP	50	56–93	74.00 (6.60)	
Heart rate	50	39–79	60.54 (7.59)	
PWV	50	9–18	12.6 (2.1)	
TMT	49	−37.01–116.03	41.04 (27.69)	
RMET (%)	48	50.0–91.7	75.2 (9.5)	
TASIT (%)	49	57.8–92.2	76.0 (8.8)	

Abbreviations: BMI = body mass index, current medication use = current antihypertensive medication use, BP = blood pressure, PWV = pulse wave velocity, TMT = Trail Making Test difference score (Part B minus Part A) used to index executive function, RMET = Reading the Mind in the Eyes Test, TASIT = The Awareness of Social Inference Test.

**Fig. 1:**
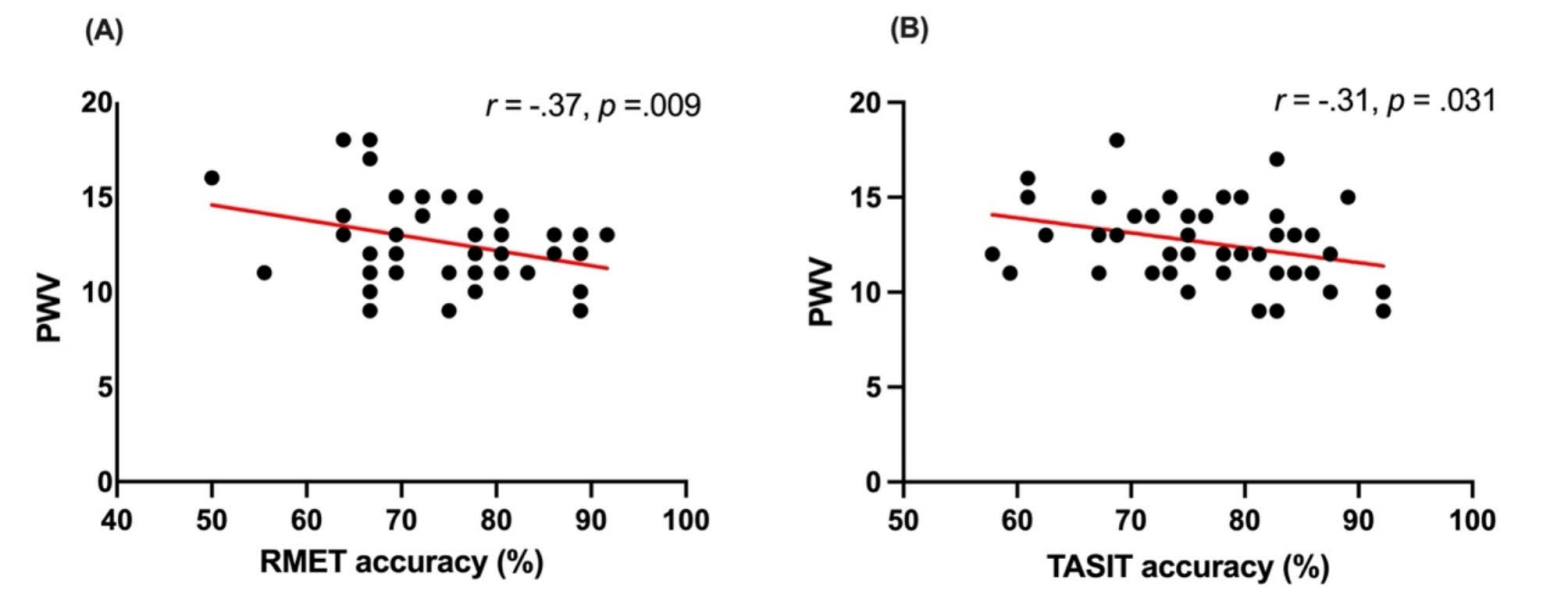
Scatterplots Illustrating the Relationship Between Pulse Wave Velocity (PWV) and (A) RMET Accuracy, and (B) TASIT Accuracy.

PWV was significantly correlated with the RMET, (*r* = − .37, *p* = .009) and TASIT, (*r* = − .31, *p* = .031). These associations are presented visually in Fig. 1. As can be seen in Table 2, the hierarchical linear regression indicated that the control variables at Step 1 did not account for significant variance in RMET performance. The inclusion of PWV at Step 2 accounted for 11% of unique variance in task scores (See Table 2). However, the final model with all predictors was not significant, *F*(6,46) = 1.32, *p* = .270. Similarly, for the TASIT, the control variables at Step 1 did not account for significant variance in task scores. At Step 2, PWV was a significant predictor of TASIT performance, accounting for an additional 9% of unique variance in task performance (see Table 3). However, the final model with all predictors was not significant, *F*(6,47) = 1.64, *p* = .160. These results were unchanged when medication use was included in the model.

**Table 2:** Hierarchical multiple regression results for the RMET.

Variable	β	t	*p*	95% CI	*R* ^2^	ΔR^2^	F (change)	*p*
Step 1					0.05	0.05	0.46	0.804
Age	− 0.17	1.09	0.283	[−1.14, 0.34]				
Sex	0.04	0.26	0.796	[−5.68, 7.36]				
Education	0.05	0.27	0.787	[−0.90,1.18]				
BMI	− 0.05	0.31	0.762	[−0.92, 0.68]				
TMT	− 0.15	0.95	0.349	[−0.17, 0.06]				
Step 2					0.17	0.11	5.38	0.026
Age	− 0.01	0.07	0.946	[−0.80, 0.75]				
Sex	− 0.08	0.45	0.653	[−7.98,5.06]				
Education	− 0.01	0.02	0.986	[−1.01,0.99]				
BMI	− 0.03	0.18	0.860	[−0.82,0.70]				
TMT	− 0.10	0.66	0.510	[−0.15,0.07]				
PWV	− 0.40	2.32	0.026	[−3.24,−0.22]				

Note. BMI = body mass index, TMT = trail making test (difference score used), PWV = pulse wave velocity.

**Table 3:** Hierarchical multiple regression results for the TASIT.

Variable	β	t	*p*	95% CI	*R* ^2^	ΔR^2^	F (change)	*p*
Step 1					0.11	0.11	1.00	0.428
Age	− 0.19	1.29	0.204	[−1.09, 0.24]				
Sex	− 0.02	0.12	0.902	[−6.26, 5.53]				
Education	− 0.15	0.93	0.356	[−1.38, 0.51]				
BMI	0.07	0.43	0.673	[−0.57, 0.88]				
TMT	0.25	1.63	0.111	[−0.02, 0.18]				
Step 2					0.19	0.09	4.43	0.041
Age	− 0.05	0.32	0.747	[−0.82, 0.59]				
Sex	− 0.11	0.68	0.499	[−7.87, 3.89]				
Education	− 0.18	1.15	0.259	[−1.43, 0.40]				
BMI	0.08	0.54	0.589	[−0.51,0.88]				
TMT	0.27	1.87	0.068	[−0.01, 0.18]				
PWV	− 0.35	2.11	0.041	[−2.79, −0.06]				

Note. BMI = body mass index, TMT = trail making test (difference score used), PWV = pulse wave velocity.

## Discussion

Age-related changes in social cognition are well established but less is known about the factors that might lead to social cognitive decline in old age. This study is the first investigation into the relationship between arterial stiffness (measured via PWV) and social cognitive aging (measured via the RMET and TASIT). In line with our predictions, PWV was significantly and moderately correlated with both measures of ToM, such that higher PWV was associated with poorer ToM performance. The regression analyses also showed PWV was a unique predictor of ToM after controlling for age, sex, education, BMI and executive function. This effect was moderate in magnitude for both tasks, with PWV accounting for 11% of the variance in RMET scores and 9% of the variance in TASIT scores. These findings add to a large literature indicating that arterial stiffness is a key predictor of cognitive aging^20^, and meaningfully extend this body of work by showing for the first time that arterial stiffness, when indexed directly via PWV, is a unique predictor of cognitive and affective ToM in late adulthood. These findings suggest that older adults who have poorer vascular health may be less sensitive to social cues and therefore may have greater difficulty understanding others in social interactions, which may have important implications for their social relationships and broader well-being.

Our findings partially support the conclusions made by Fischer et al.^31,32^ who identified small but significant associations between cognitive ToM and pulse pressure. However, unlike our study, they failed to find to a relationship between pulse pressure and affective ToM (i.e., performance on the RMET). As noted earlier, pulse pressure is often used as a surrogate for arterial stiffness but its measurement can be affected by other factors, such as stroke volume^33,34^. These findings suggest that when arterial stiffness is measured directly using carotid-femoral PWV, it is sensitive in predicting both cognitive and affective ToM in older age.

While this study has demonstrated that arterial stiffness and social cognition are related in older age, the precise mechanism underlying this relationship remains unclear. However, one potential explanation may be white matter damage. Prior work has shown a relationship between increased cerebral arterial stiffness and poorer cognition, that is mediated by white matter lesions^43,44^, and there is some evidence that white matter hyperintensities are associated with social cognition in older adults^45^. Other work has also shown that increased arterial stiffness disrupts network organization, reducing cognitive efficiency^46^. Future studies should aim to extend on our study by testing whether there are any important mediators of the relationship between arterial stiffness and social cognition.

Another important step in this literature will be establishing whether there are any moderators of this relationship. Indeed, recent work has shown that diet may moderate the relationship between arterial stiffness and cognitive function in healthy older adults with subjective memory complaints^47^. Diet has also been linked to social cognitive abilities in older age^48^. Other work has shown that demographic factors, such as sex, may moderate the association between stiffness and cognition in older adults^49,50^. Given that sex differences have been identified in social cognitive processing^51^ and that the rate of arterial stiffening in older age differs as a function of sex^52^, it will be important to establish whether sex also moderates the relationship between stiffness and social cognition in older adults. Our prior work demonstrated that cardiorespiratory fitness and muscular strength were not associated with social cognitive function in older age^41^. However, it is possible that these variables may be important moderators of the relationship between stiffness and social cognition. This is an important question for future research with larger sample sizes with sufficient power for moderation analyses.

While this study was the first to show an association between arterial stiffness and social cognition, there are limitations that need to be acknowledged, as well as several important directions for future research. Firstly, we only included healthy older adult participants in this study, but prior work has shown that the relationship between vascular aging and cognition may be greater in groups that have known vascular impairments^45^. Second, our sample was sufficiently powered to detect a moderate effect but was not large enough to run moderation analyses. Considering these limitations, future work is now needed with larger and more diverse samples that include healthy older adults as well as those with vascular risk factors. Third, we used the RMET because it is arguably the most widely used measure of ToM, particularly in the aging literature^12^. However, concerns regarding the psychometric properties of the RMET have been raised recently^53^, and therefore these findings should be interpreted with some degree of caution. Fourth, our study was restricted to a single domain of social cognition (i.e., ToM) but there are other domains of social cognition, which also change with normal aging that warrant investigation. Therefore, we would encourage future researchers to build on this work by including other validated measures of ToM, as well as measures indexing other domains of social cognition, such as emotion perception. Finally, this study used a cross-sectional design, and although this is a limitation of the social cognitive aging literature more broadly, it does limit the conclusions that can be drawn from this dataset. Specifically, it is not possible to infer causality, nor is it possible to establish when during the lifespan arterial stiffness starts to predict social cognitive performance, or whether this relationship is in fact bidirectional. Indeed, people who have poorer physical health may be less likely to go out and engage their social cognitive abilities, which may lead to social cognitive decline. Future work should aim to examine the role arterial stiffness plays in social cognition using longitudinal assessments with large lifespan samples.

 To conclude, this study has shown for the first time that arterial stiffness, measured via PWV, is a unique predictor of social cognition in healthy older adults. These findings indicate that people who have poorer vascular health may also have greater difficulties understanding how other people think and feel, which has important implications for social functioning and broader well-being. Given that the rate of arterial stiffness can be modified with lifestyle changes, these findings may have important implications for preventative approaches for maintaining social wellbeing in older age.

## Data Availability

All data are available by request from the first author (S.G).

## References

[1] Krendl, A. C., Kennedy, D. P., Hugenberg, K. & Perry, B. L. Social Cognitive abilities Predict Unique aspects of older adults’ personal Social Networks. *J. Gerontol. B Psychol. Sci. Soc. Sci.* **77**, 18–28 (2022). 33733655 10.1093/geronb/gbab048 PMC8755914

[2] Hein, G. et al. A social information processing perspective on social connectedness. *Neurosci. Biobehav Rev.* **167**, 105945. 10.1016/j.neubiorev.2024.105945 (2024). 39549980 10.1016/j.neubiorev.2024.105945

[3] Holt-Lunstad, J. Loneliness and social isolation as risk factors: the power of social connection in Prevention. *Am. J. Lifestyle Med.* **15**, 567–573 (2021). 34646109 10.1177/15598276211009454 PMC8504333

[4] Luo, Y., Hawkley, L. C., Waite, L. J. & Cacioppo, J. T. Loneliness, health, and mortality in old age: a national longitudinal study. *Soc. Sci. Med.* **74**, 907–914 (2012). 22326307 10.1016/j.socscimed.2011.11.028 PMC3303190

[5] Steptoe, A., Shankar, A., Demakakos, P. & Wardle, J. Social isolation, loneliness, and all-cause mortality in older men and women. *Proc. Natl. Acad. Sci. U S A*. **110**, 5797–5801 (2013). 23530191 10.1073/pnas.1219686110 PMC3625264

[6] Lennartsson, C., Rehnberg, J. & Dahlberg, L. The association between loneliness, social isolation and all-cause mortality in a nationally representative sample of older women and men. *Aging Ment Health*. **26**, 1821–1828 (2022). 34550832 10.1080/13607863.2021.1976723

[7] Samtani, S. et al. Associations between social connections and cognition: a global collaborative individual participant data meta-analysis. *Lancet Health Longev.* **3**, E740–E. 10.1016/S2666-7568(22)00199-4 (2022). 10.1016/S2666-7568(22)00199-4 PMC9750173 36273484

[8] Mahalingam, G. et al. Social connections and risk of incident mild cognitive impairment, dementia, and mortality in 13 longitudinal cohort studies of ageing. *Int. Psychogeriatr.* **35**, 16–17 (2023). 10.1002/alz.13072 PMC10603208 37102417

[9] Henry, J. D., von Hippel, W., Molenberghs, P., Lee, T. & Sachdev, P. Clinical assessment of social cognitive function in neurological disorders. *Nat. Rev. Neurol.* **12**, 28–39 (2016). 26670297 10.1038/nrneurol.2015.229

[10] Grainger, S. A. et al. Aging is Associated with multidirectional changes in Social Cognition: findings from an adult life-span sample ranging from 18 to 101 years. *J. Gerontol. B Psychol. Sci. Soc. Sci.* **78**, 62–72 (2023). 35985278 10.1093/geronb/gbac110 PMC9890910

[11] McKay, K. T., Talipski, L. A., Grainger, S. A., Alister, M. & Henry, J. D. How does Aging affect social attention? A test of competing theories using Multilevel Meta-Analysis. *J. Gerontol. B Psychol. Sci. Soc. Sci.* **77**, 1454–1463 (2022). 35279031 10.1093/geronb/gbac052 PMC9371458

[12] Henry, J. D., Phillips, L. H., Ruffman, T. & Bailey, P. E. A meta-analytic review of age differences in theory of mind. *Psychol. Aging*. **28**, 826–839 (2013). 23276217 10.1037/a0030677

[13] Shamay-Tsoory, S. G. & Aharon-Peretz, J. Dissociable prefrontal networks for cognitive and affective theory of mind: a lesion study. *Neuropsychologia* **45**, 3054–3067 (2007). 17640690 10.1016/j.neuropsychologia.2007.05.021

[14] Grainger, S. A., Steinvik, H. R., Henry, J. D. & Phillips, L. H. The role of social attention in older adults’ ability to interpret naturalistic social scenes. *Q. J. Exp. Psychol. (Hove)*. **72**, 1328–1343 (2019). 30001675 10.1177/1747021818791774

[15] Phillips, L. H. et al. Lifespan aging and belief reasoning: influences of executive function and social cue decoding. *Cognition* **120**, 236–247 (2011). 21624567 10.1016/j.cognition.2011.05.003

[16] Hughes, M. L., Agrigoroaei, S., Jeon, M., Bruzzese, M. & Lachman, M. E. Change in cognitive performance from midlife Into Old Age: findings from the midlife in the United States (MIDUS) Study. *J. Int. Neuropsychol. Soc.* **24**, 805–820 (2018). 30019663 10.1017/S1355617718000425 PMC6170692

[17] Wolters, F. J. et al. Cerebral perfusion and the risk of dementia: a Population-based study. *Circulation* **136**, 719–728 (2017). 28588075 10.1161/CIRCULATIONAHA.117.027448

[18] van Sloten, T. T. et al. D. A. Association between arterial stiffness, cerebral small vessel disease and cognitive impairment: a systematic review and meta-analysis. *Neurosci. Biobehavioral Reviews*. **53**, 121–130 (2015). 10.1016/j.neubiorev.2015.03.011 PMC5314721 25827412

[19] Hazzouri, Z. A. Pulse wave velocity and cognitive decline in elders: the Health, Aging, and body composition study. *Stroke* **44**, 388–393 (2013). 23321445 10.1161/STROKEAHA.112.673533 PMC3572783

[20] Alvarez-Bueno, C. et al. Arterial stiffness and cognition among adults: a systematic review and Meta-analysis of Observational and Longitudinal studies. *J. Am. Heart Assoc.* **9**, e014621. 10.1161/JAHA.119.014621 (2020). 32106748 10.1161/JAHA.119.014621 PMC7335587

[21] Henry, J. D., von Hippel, W. & Baynes, K. Social inappropriateness, executive control, and aging. *Psychol. Aging*. **24**, 239–244 (2009). 19290759 10.1037/a0013423

[22] Otsuka, Y., Shizawa, M., Sato, A. & Itakura, S. The role of executive functions in older adults’ affective theory of mind. *Arch. Gerontol. Geriatr.* **97**, 104513. 10.1016/j.archger.2021.104513 (2021). 34481137 10.1016/j.archger.2021.104513

[23] Amodio, D. M. & Frith, C. D. Meeting of minds: the medial frontal cortex and social cognition. *Nat. Rev. Neurosci.* **7**, 268–277 (2006). 16552413 10.1038/nrn1884

[24] Yuan, P. & Raz, N. Prefrontal cortex and executive functions in healthy adults: a meta-analysis of structural neuroimaging studies. *Neurosci. Biobehav Rev.* **42**, 180–192 (2014). 24568942 10.1016/j.neubiorev.2014.02.005 PMC4011981

[25] Iadecola, C. & Davisson, R. L. Hypertension and cerebrovascular dysfunction. *Cell. Metab.* **7**, 476–484 (2008). 18522829 10.1016/j.cmet.2008.03.010 PMC2475602

[26] Palta, P. et al. Central arterial stiffness is Associated with Structural Brain damage and poorer cognitive performance: the ARIC Study. *J. Am. Heart Association: Cardiovasc. Cerebrovasc. Disease* **8** e011045. https://doi.org/10.1161/JAHA.118.011045 (2019). 10.1161/JAHA.118.011045 PMC6497348 30646799

[27] Climie, R. E. et al. Measuring the Interaction between the macro- and micro-vasculature. *Front. Cardiovasc. Med.* **6**, 169 (2019). 31824963 10.3389/fcvm.2019.00169 PMC6882776

[28] Singer, J., Trollor, J. N., Baune, B. T., Sachdev, P. S. & Smith, E. Arterial stiffness, the brain and cognition: a systematic review. *Ageing Res. Rev.* **15**, 16–27 (2014). 24548924 10.1016/j.arr.2014.02.002

[29] Ashor, A. W., Siervo, M., Lara, J., Oggioni, C. & Mathers, J. C. Antioxidant vitamin supplementation reduces arterial stiffness in adults: a systematic review and meta-analysis of randomized controlled trials. *J. Nutr.* **144**, 1594–1602 (2014). 25098780 10.3945/jn.114.195826

[30] Petersen, K. S., Blanch, N., Keogh, J. B. & Clifton, P. M. Effect of weight loss on pulse wave velocity: systematic review and meta-analysis. *Arterioscler. Thromb. Vasc Biol.* **35**, 243–252 (2015). 25414255 10.1161/ATVBAHA.114.304798

[31] Fischer, A. L., Bernstein, D. M. & Thornton, W. L. Vascular health modifies theory of mind performance in older adults. *J. Gerontol. B Psychol. Sci. Soc. Sci.* **69**, 219–227 (2014). 23325503 10.1093/geronb/gbs120

[32] Fischer, A. L., O’Rourke, N. & Loken Thornton, W. Age differences in cognitive and affective theory of mind: concurrent contributions of neurocognitive performance, sex, and pulse pressure. *J. Gerontol. B Psychol. Sci. Soc. Sci.* **72**, 71–81 (2017). 27503390 10.1093/geronb/gbw088

[33] Alfie, J., Waisman, G. D., Galarza, C. R. & Camera M. I. Contribution of stroke volume to the change in pulse pressure pattern with age. *Hypertension* **34**, 808–812 (1999). 10523365 10.1161/01.hyp.34.4.808

[34] Townsend, R. R. et al. Recommendations for improving and standardizing Vascular Research on arterial stiffness: a Scientific Statement from the American Heart Association. *Hypertension* **66**, 698–722 (2015). 26160955 10.1161/HYP.0000000000000033 PMC4587661

[35] Kim, H. L. Arterial stiffness and hypertension. *Clin. Hypertens.* **29**, 31 (2023). 38037153 10.1186/s40885-023-00258-1 PMC10691097

[36] Kim, Y. S., Kim, D. H., Choi, B. H., Sohn, E. H. & Lee, A. Y. Relationship between brachial-ankle pulse wave velocity and cognitive function in an elderly community-dwelling population with metabolic syndrome. *Arch. Gerontol. Geriatr.* **49**, 176–179 (2009). 18786736 10.1016/j.archger.2008.07.004

[37] Hsieh, S. et al. The Mini-addenbrooke’s cognitive examination: a new assessment tool for dementia. *Dement. Geriatr. Cogn. Disord*. **39**, 1–11 (2015). 25227877 10.1159/000366040 PMC4774042

[38] Baron-Cohen, S., Wheelwright, S., Hill, J., Raste, Y. & Plumb, I. The reading the mind in the eyes test revised version: a study with normal adults, and adults with Asperger syndrome or high-functioning autism. *J. Child. Psychol. Psychiatry*. **42**, 241–251 (2001). 11280420

[39] McDonald, S. et al. Reliability and validity of the awareness of Social Inference Test (TASIT): a clinical test of social perception. *Disabil. Rehabil*. **28**, 1529–1542 (2006). 17178616 10.1080/09638280600646185

[40] Sanchez-Cubillo, I. et al. Construct validity of the trail making test: role of task-switching, working memory, inhibition/interference control, and visuomotor abilities. *J. Int. Neuropsychol. Soc.* **15**, 438–450 (2009). 19402930 10.1017/S1355617709090626

[41] Grainger, S. A. et al. Cardiorespiratory Fitness and muscular strength do not predict Social Cognitive Capacity in Older Age. *J. Gerontol. B Psychol. Sci. Soc. Sci.* **78**, 1824–1833 (2023). 37480568 10.1093/geronb/gbad101 PMC10645310

[42] dos Santos, T. T. B. A. et al. The relationship between Social Cognition and Executive functions in Alzheimer’s Disease: a systematic review. *Curr. Alzheimer Res.* **17**, 487–497 (2020). 32589558 10.2174/1567205017666200626205154

[43] Bowie, D. C. et al. Neurovascular mechanisms of cognitive aging: sex-related differences in the average progression of arteriosclerosis, white matter atrophy, and cognitive decline. *Neurobiol. Dis.* **201**, 106653. 10.1016/j.nbd.2024.106653 (2024). 39214337 10.1016/j.nbd.2024.106653 PMC12184324

[44] Tan, C. H. et al. Optical measures of cerebral arterial stiffness are associated with white matter signal abnormalities and cognitive performance in normal aging. *Neurobiol. Aging*. **84**, 200–207 (2019). 31500910 10.1016/j.neurobiolaging.2019.08.004 PMC7159038

[45] Kynast, J. et al. White matter hyperintensities associated with small vessel disease impair social cognition beside attention and memory. *J. Cereb. Blood Flow. Metab.* **38**, 996–1009 (2018). 28685621 10.1177/0271678X17719380 PMC5999004

[46] Kong, T. S. et al. Age-related differences in functional brain network segregation are consistent with a cascade of cerebrovascular, structural, and cognitive effects. *Netw. Neurosci.* **4**, 89–114 (2020). 32043045 10.1162/netn_a_00110 PMC7006874

[47] Gauci, S. et al. Diet May Moderate the Relationship between arterial stiffness and cognitive performance in older adults. *J. Alzheimers Dis.* **85**, 815–828 (2022). 34864661 10.3233/JAD-210567 PMC8842781

[48] Ruffman, T., Zhang, J., Taumoepeau, M. & Skeaff, S. Your way to a better theory of mind: a healthy Diet relates to Better Faux Pas Recognition in older adults. *Exp. Aging Res.* **42**, 279–288 (2016). 27070046 10.1080/0361073X.2016.1156974

[49] Dao, E., Barha, C. K., Santos, M., Welch, M. & Liu-Ambrose, T. Sex differences in the relationship between arterial stiffness and cognitive function in older adults. *J. Stroke Cerebrovasc. Dis.* **31**, 106175. 10.1016/j.jstrokecerebrovasdis.2021.106175 (2022). 34715522 10.1016/j.jstrokecerebrovasdis.2021.106175

[50] Sabra, D. et al. Sex moderations in the relationship between aortic stiffness, cognition, and cerebrovascular reactivity in healthy older adults. *PLoS One*. **16**, e0257815. 10.1371/journal.pone.0257815 (2021). 34582484 10.1371/journal.pone.0257815 PMC8478243

[51] Greenberg, D. M. et al. Sex and age differences in theory of mind across 57 countries using the English version of the reading the mind in the eyes test. *P Natl. Acad. Sci. USA*. **120**, e2022385119. 10.1073/pnas.2022385119 (2022). 10.1073/pnas.2022385119 PMC9910622 36584298

[52] Zaydun, G. et al. Menopause is an independent factor augmenting the age-related increase in arterial stiffness in the early postmenopausal phase. *Atherosclerosis* **184**, 137–142 (2006). 15913634 10.1016/j.atherosclerosis.2005.03.043

[53] Higgins, W. C., Ross, R. M., Langdon, R. & Polito, V. The reading the mind in the eyes test shows poor Psychometric properties in a large, demographically Representative U.S. Sample. *Assessment* **30**, 1777–1789 (2023). 36124391 10.1177/10731911221124342

